# Genomic Epidemiology and Lineage Dynamics of SARS-CoV-2 in Bulgaria: Insights from a Three-Year Pandemic Analysis

**DOI:** 10.3390/v15091924

**Published:** 2023-09-15

**Authors:** Marta Giovanetti, Eleonora Cella, Ivan Ivanov, Lyubomira Grigorova, Ivan Stoikov, Deyan Donchev, Reneta Dimitrova, Svetoslav Nanev Slavov, Carla Mavian, Vagner Fonseca, Fabio Scarpa, Alessandra Borsetti, Neli Korsun, Ivelina Trifonova, Veselin Dobrinov, Todor Kantardjiev, Iva Christova, Massimo Ciccozzi, Ivailo Alexiev

**Affiliations:** 1 Instituto Rene Rachou Fundação Oswaldo Cruz, Belo Horizonte 30190-009, Brazil; 2 Sciences and Technologies for Sustainable Development and One Health, Università Campus Bio-Medico di Roma, 00128 Rome, Italy; 3 Climate Amplified Diseases and Epidemics (CLIMADE), Brazil; 4 Burnett School of Biomedical Sciences, University of Central Florida, Orlando, FL 32816, USA; 5 National Center of Infectious and Parasitic Diseases, 1504 Sofia, Bulgaria; ivanoov@gmail.com (I.I.); lyubomiragrigorova@gmail.com (L.G.); ivanstoikovbt@gmail.com (I.S.); didoneobux93@abv.bg (D.D.); naydenova.reneta@gmail.com (R.D.); neli_korsun@abv.bg (N.K.); trifonova.ivelina@abv.bg (I.T.); veselin8d8@abv.bg (V.D.); todorkantardjiev@gmail.com (T.K.); iva_christova@yahoo.com (I.C.); ivoalexiev@yahoo.com (I.A.); 6 Butantan Institute, São Paulo 05508-040, Brazil; svetoslav.slavov@hemocentro.fmrp.usp.br; 7 Blood Center of Ribeirão Preto, Faculty of Medicine of Ribeirão Preto, University of São Paulo, Ribeirão Preto 14051-140, Brazil; 8 Emerging Pathogens Institute, Department of Pathology, Immunology and Laboratory Medicine, College of Medicine, University of Florida, Gainesville, FL 32610, USA; cmavian@ufl.edu; 9 Department of Exact and Earth Sciences, University of the State of Bahia, Salvador 40285-001, Brazil; vagnerfonseca@gmail.com; 10 Coordenação de Vigilância, Preparação e Resposta à Emergências e Desastres (PHE), Organização Pan-Americana da Saúde/Organização Mundial da Saúde (OPAS/OMS), Brasilia 70312-970, Brazil; 11 Department of Biomedical Sciences, University of Sassari, 07100 Sassari, Italy; fscarpa@uniss.it; 12 National HIV/AIDS Research Center (CNAIDS), National Institute of Health, 00118 Rome, Italy; alessandra.borsetti@iss.it; 13 Unit of Medical Statistics and Molecular Epidemiology, Universita Campus Bio-Medico di Roma, 00128 Rome, Italy; m.ciccozzi@unicampus.it

**Keywords:** Bulgaria, SARS-CoV-2, genomic surveillance, viral evolution, epidemiology

## Abstract

The COVID-19 pandemic caused by severe acute respiratory syndrome coronavirus 2 (SARS-CoV-2) has brought about significant challenges worldwide. In this study, we present a comprehensive analysis of the genomic epidemiology and lineage dynamics of SARS-CoV-2 in Bulgaria over a three-year period. Through extensive genomic sequencing and data analysis, we investigated the evolution of the virus, the emergence of variants of concern (VOCs), and their impact on the country’s pandemic trajectory. We also assessed the relationship between viral diversity and COVID-19 morbidity and mortality in Bulgaria. Our findings shed light on the temporal and spatial distribution of SARS-CoV-2 lineages and provide crucial insights into the dynamics of the pandemic in the country. The interplay between international travel and viral transmission plays a significant role in the emergence and dissemination of different SARS-CoV-2 variants. The observed proportions of exportation to various continents provide insights into the potential pathways through which these lineages spread globally. Understanding the genomic epidemiology of SARS-CoV-2 in Bulgaria is essential for formulating targeted public health strategies, enhancing vaccination efforts, and effectively managing future outbreaks.

## 1. Introduction

In December 2019, the severe acute respiratory syndrome coronavirus 2 (SARS-CoV-2) first emerged in Wuhan, China, and rapidly disseminated across the globe, giving rise to the most significant respiratory infection since the 1918 influenza outbreak. Consequently, extensive modifications were required in health systems worldwide, along with the implementation of comprehensive diagnostic and viral genome sequencing programs. These global initiatives proved essential for enabling informed decision-making and implementing effective health policies in response to the pandemic’s challenges [1,2].

One critical aspect that significantly impacted the course of the pandemic was the emergence of SARS-CoV-2 with specific point mutations occurring at key sites. These mutations led to the development of VOCs, which exhibited increased infectivity and transmission potential [3,4]. Subsequently, these VOCs caused epidemic waves in different countries around the world, with the response varying depending on the undertaken public health measures and vaccination rates in each country. This variability in the response to the pandemic resulted in differing morbidity and mortality rates [5,6].

Eastern Europe, notably Bulgaria, experienced a considerably high COVID-19-related mortality rate [7]. During the initial stages of the pandemic, Bulgaria implemented control policies that initially helped to manage the pandemic’s progression. However, subsequent epidemic waves severely impacted the country, leading to one of the highest mortality rates in the world, exceeding 1% when considering its population of approximately 7 million. Surprisingly, while Western Europe witnessed a decline in COVID-19-related deaths, Bulgaria continued to suffer from significant morbidity and mortality due to the Omicron variant’s emergence [8,9]. Notably, COVID-19 vaccination rates in Eastern Europe have been consistently lower than those in Western Europe, and specifically in Bulgaria, there has been low vaccination coverage, with only 30% of the country immunized [8].

Despite the efforts of the government to control the spread of the virus and the large-scale sequencing of SARS-CoV-2 at the National Center of Infectious and Parasitic Diseases, the genomic diversity and evolution of circulating SARS-CoV-2 lineages in Bulgaria have not been studied in detail [10]. Although there are over 21 thousand SARS-CoV-2 sequences from Bulgaria available in public databases such as GISAID, comprehensive information about the virus’s evolution and the impact of VOCs remains elusive. To address these needs, the present study aims to shed light on the diversity of SARS-CoV-2 during the three-year period encompassing the pandemic and its epidemic waves and its impact on the health system of the country. Moreover, this study offers valuable insights into the potential factors that may have contributed to Bulgaria’s viral progression during the pandemic by examining the evolution and substitution of SARS-CoV-2 variants over time, as well as the influence of importation and exportation events to and from the country.

Understanding these critical factors is pivotal for the development of more effective and focused public health measures and enhanced outbreak management strategies in the future. By identifying key drivers behind the higher number of notified cases and deaths, this study could help inform policymakers and healthcare authorities to implement targeted interventions, mitigate the impact of future pandemics, and improve public health in the country.

## 2. Materials and Methods

### 2.1. Sequence Data Collection

To conduct a comprehensive investigation of SARS-CoV-2’s genomic epidemiology in Bulgaria, we retrieved all available Bulgarian full-length viral genomes from the GISAID database up to 31 March 2023 (https://www.gisaid.org/). Only genomes > 29,000 bp and with <1% ambiguities were considered. Quality assessment was performed to exclude incomplete or problematic sequences and those with incomplete metadata. Specifically, we used the NextClade analysis pipeline [11] to identify and exclude sequences that had missing genomic regions (>10%), ambiguous bases (>10), or sequences flagged with clustered mutations by NextClade. Additionally, we aligned the Bulgarian datasets with globally representative sequences to evaluate the virus spread in Bulgaria. The lineage classification was carried out using the dynamic lineage classification protocol specified in the Phylogenetic Assignment of Named Global Outbreak LINeages (Pangolin) version 4.1.3 [12]. To mitigate sampling biases, we carefully selected genomic sequences to ensure that the sampled datasets broadly reflected global reported case counts. To achieve this, we employed the subsampler tool (https://github.com/andersonbrito/subsampler (accessed on 1 September 2023)) [13], which allowed us to subsample sequences per country based on case counts over the study period. By using this approach, we aimed to create geographically, temporally, and epidemiologically representative datasets by focusing on the VOCs which led the epidemic progression in the country (Alpha, Delta, Omicron BA.1x, Omicron BA.2x and Omicron BA.5x). To ensure a robust and comprehensive representation for analysis, each VOC’s dataset comprised approximately 20,000 sequences. The final datasets for each VOC were as follows: (i) Alpha (n = 13,010), including 3083 Bulgarian genomes; (ii) Delta (n = 20,896), including 10,290 Bulgarian genomes; (iii) Omicron BA.1.x (n = 13,958), including 4163 Bulgarian genomes; (iv) Omicron BA.2.x (n = 11,925), including 1703 Bulgarian genomes; and (v) Omicron BA.5.x (n = 11,026), including 709 Bulgarian genomes.

### 2.2. Phylogenetic Analysis

The sequences were initially aligned using ViralMSA with default parameters [14]. Subsequently, manual curation was carried out using Aliview [15] to eliminate artifacts within the alignment and terminal regions, ensuring data integrity. Phylogenetic analysis was then conducted using the maximum likelihood method implemented in IQ-TREE v.2 [16]. The tree was inferred using the general time reversible (GTR) model of nucleotide substitution and a proportion of invariable sites (+I), determined by the ModelFinder application. The branch support was assessed via the approximate likelihood-ratio test based on the bootstrap and the Shimodaira–Hasegawa-like procedure (SH-aLRT) with 1000 replicates. Subsequently, the maximum likelihood trees were transformed into time-scaled phylogenies using TreeTime [17], employing a standard mutation rate of 0.0008 substitutions per site per year and a standard clock deviation of 0.0004. Outlier sequences that deviated from the strict molecular clock assumption, as identified by TreeTime, were systematically removed with the Ape package in R [18] until achieving an optimal time-scaled phylogeny. For each VOC, we generated time-scaled tree topologies and performed discrete ancestral state reconstruction, specifically regarding geographic locations. This reconstruction allowed us to infer the global dissemination of each variant using the migration package extension of TreeTime under a GTR model. Finally, utilizing a custom Python script, we counted the number of state changes by iterating over each phylogeny from the root to the external tips. State changes were registered when an internal node transitioned from one country to a different country in the resulting child node or tip(s), thus indicating importation or exportation events. The timing of these transition events was recorded, providing estimated information on importation and exportation events. All results were visualized using the ggtree ggplot libraries [19,20].

### 2.3. Epidemiology Data Assembly

To complement the genomic analysis, COVID-19 cases and the death count data for Bulgaria were obtained from the Our World in Data repository (https://github.com/owid/covid-19-data/tree/master/public/data) (up to 31 March 2023). The information regarding sampling dates and viral lineages was compiled from the available metadata in the GISAID database.

## 3. Results

### 3.1. Key Milestones of the SARS-CoV-2 Pandemic in Bulgaria

**Figure 1:**
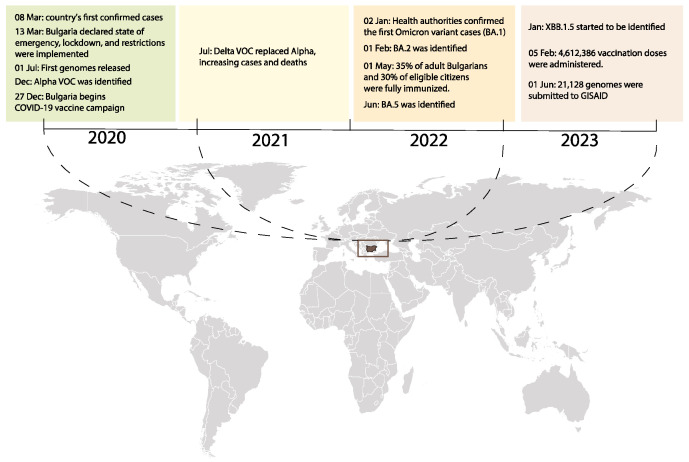
Timeline of SARS-CoV-2 key events in Bulgaria. This timeline summarizes key events in the SARS-CoV-2 pandemic in Bulgaria, offering an overview of critical milestones and trends throughout the pandemic period. The following major events occurred: (1) the first confirmed cases; (2) the implementation of public health measures; (3) waves of infections; (d4 vaccination campaigns; (5) the emergence of VOCs; and (6) milestones in research and healthcare.

The emergence of the first two confirmed cases of SARS-CoV-2 infection in Bulgaria on 8 March 2020 marked the beginning of the nation’s battle against the pandemic. These cases involved a 27-year-old man from Pleven and a 75-year-old woman from Gabrovo. Notably, these patients did not report any travel history to known coronavirus hotspots, suggesting that their infections were likely acquired locally. This raised nationwide concern regarding the capacity to quickly contain the viral spread, as it indicated the presence of community transmission (Figure 1).

As the number of COVID-19 patients steadily increased, the Bulgarian government responded with swift and decisive action, declaring a state of emergency on 13 March (Figure 1). This period was marked by the implementation of various containment measures to limit the spread of the virus and protect public health. Among these measures was a 14-day preventive house quarantine for citizens who had been in contact with a COVID-19 patient or had returned from high-risk regions. In addition, patients who tested positive for the virus underwent a 21-day house quarantine. This quarantine period lasted until a subsequent negative test confirmed their recovery. The implementation of these containment measures aimed to reduce the transmission of the virus and alleviate the strain on the healthcare system. By isolating potentially infected individuals and closely monitoring the recovery of patients, the government sought to break the chain of transmission and prevent further escalation of the pandemic.

The evolving nature of the virus required flexibility in Bulgaria’s response strategy. The National Crisis-Management Staff considered the World Health Organization’s findings, and as a result, extended the recovery house quarantine to 28 days. Following the WHO’s declaration of the outbreak as a public health emergency of international concern on 30 January 2020, the Bulgarian government introduced a series of additional restriction measures (Figure 1). Social isolation measures were among the primary strategies implemented, along with the closure of schools, universities, and non-essential businesses. The mandatory use of personal protective masks, the cancellation of large gatherings and events, and the limited opening of essential services, such as markets and pharmacies, were also implemented. As the pandemic progressed, restrictions were gradually eased to alleviate the adverse impact on the economy. Despite implementing these measures, the daily increase in COVID-19 cases persisted, and in some instances, it was linked to the detection of variants of concern such as Alpha, Delta, and later, Omicron strains (Figure 1). Similarly, recombinant forms were detected globally, and subsequently, they were also detected in Bulgaria. This challenging situation led the Bulgarian government to take decisive action aimed at maintaining necessary control and management over the pandemic, as the authorities grappled with the evolving nature of the virus and its impact on public health. Throughout this critical period, vaccination efforts played a pivotal role in Bulgaria’s response to the pandemic (Figure 1). As of 5 February 2023, a total of 4,612,386 vaccine doses had been administered in the country. This achievement translated to 12.17 individuals being vaccinated with at least one booster or additional dose per 100 people, as illustrated in Figure 1. However, it is worth noting that this rate appeared to be lower than the global average, estimated at 31.8 individuals per 100 people [21]. Despite this disparity, these vaccinations represented a significant milestone in the nation’s ongoing battle against the virus, instilling hope for a brighter future and bolstering efforts to safeguard the health and well-being of the population.

### 3.2. SARS-CoV-2 Pandemic History and Variant Displacement in Bulgaria

**Figure 2:**
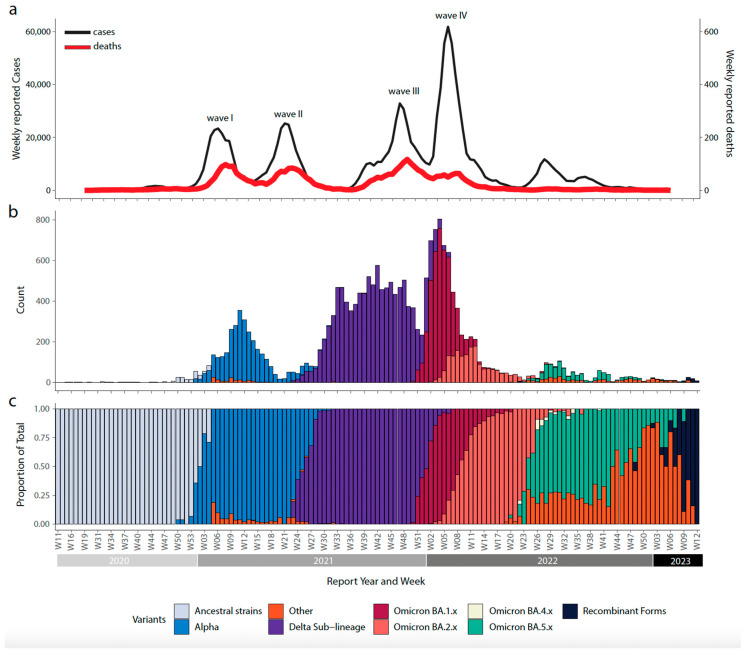
SARS-CoV-2 epidemic dynamics in Bulgaria. (**a**) Daily count of COVID-19 cases and associated deaths in Bulgaria over time (**Upper**); (**b**) distribution of sequenced isolates of SARS-CoV-2 in Bulgaria, categorized by different variants (**Middle**); (**c**) progression in the proportion of circulating variants in Bulgaria over the different waves of infection, showing the rapid replacement of different VOCs throughout time (**bottom**).

By the end of 2020, Bulgaria experienced one of the highest pandemic-related excess mortality rates globally, despite initially being recognized as a containment success story during the early wave that heavily impacted Western Europe and the Americas. Nevertheless, the COVID-19 pandemic in Bulgaria unfolded in four distinct waves from 2021 to 2023, marked by a significant reduction in total cases and deaths, due to the gradual accumulation of herd immunity since the onset of the pandemic (Figure 2).

The initial wave emerged in late 2020, primarily driven by ancestral strains of the virus, resulting in 193,274 cases and 8458 deaths (Figure 2). However, the situation changed in early 2021 with the emergence of the Alpha variant, a variant of concern with specific point mutations that increased its transmissibility and disease severity (Figure 2). This variant accounted for 20,208 cases and 1769 deaths. Following a period of relative stability, a third wave emerged towards the end of 2021, fueled by the rapid spread of the Delta variant, which entirely displaced the previously circulating variant of concern in June–July 2021 (Figure 2). The Delta variant was responsible for 231,972 cases and 9598 deaths. The dominance of the Delta variant persisted until the end of 2021. During this period, the distribution of vaccines, which began on 27 December 2020, played a crucial role in the response to the pandemic, aiming to strengthen the defense against the virus in the country and contribute to the accumulation of herd immunity.

In early 2022, Bulgaria faced the emergence of the fourth wave of infections, triggered by the emergence of the Omicron strains (Figure 2), contributing to 409,035 cases and 6200 deaths. The Omicron BA.1 variant displaced the Delta variant in January 2022, marking the beginning of this fourth main wave. The BA.1 wave had a profound impact, resulting in a notable increase in the number of cases and a novel peak in the death toll. This situation marked Bulgaria as the first country where COVID-19-related excess deaths exceeded 1% of the total population [8]. This critical situation prompted the implementation of robust public health measures and a proactive approach to mitigate the consequences of the pandemic on the population. Later, there were subsequent minor waves characterized by the prevalence of the Omicron BA.2 and Omicron BA.5 lineages, with BA.2 emerging by the end of March and BA.5 by June 2022. These developments marked the commencement of the endemic phase and initiated new cycles of variant displacement. The coexistence of different lineages led to the emergence of recombinant forms of the virus. Towards the end of 2022, these first recombinant forms were identified, marking a significant shift in the virus’s evolution (Figure 2). Throughout 2023, these recombinant forms gradually gained prominence and eventually became the predominant lineages by March 2023. The widespread transmission and prevalence of these recombinant variants posed new challenges for public health authorities, necessitating intensified efforts to monitor and respond to their impact on the population’s health.

### 3.3. Phylogenetic Inference and Viral Dissemination Patterns of SARS-CoV-2 in Bulgaria

**Figure 3:**
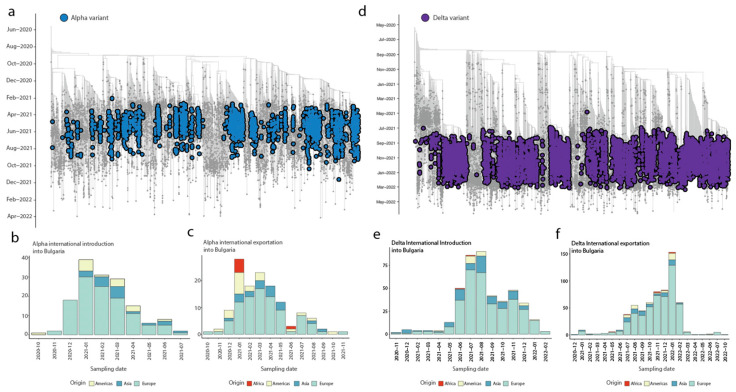
Inferred viral dissemination patterns of Alpha and Gamma variant of concerns (VOCs) in Bulgaria. Panels (**a**,**d**) represent dated phylogenies of Alpha and Delta lineages, respectively, observed in Bulgaria. Panels (**b**–**f**) show the inferred viral exchange patterns involving Bulgaria for the two VOCs. Viral introductions and exportations are color-coded based on their continent of origin or destination.

**Figure 4:**
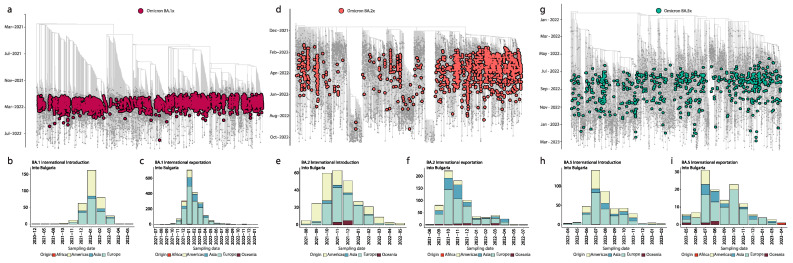
Inferred viral dissemination patterns of Omicron variants of concern (VOCs) in Bulgaria. Panels (**a**,**d**,**g**) represent dated phylogenies of Omicron BA.1.x, BA.2.x, and BA.5.x lineages, respectively, observed in Bulgaria. Panels (**b**,**c**,**e**,**f**,**h**,**i**) show the inferred viral exchange patterns involving Bulgaria for the three VOCs. Viral introductions and exportations are color-coded based on their continent of origin or destination.

To understand the dynamics of SARS-CoV-2 spread in Bulgaria, our analysis focused on the main variants of concern (Alpha, Delta, Omicron BA.1, Omicron BA.2, and Omicron BA.5) that fueled the epidemic situation in the country. By constructing a date-stamped phylogeny for each VOC (Figure 3a,d and Figure 4a,d,g), we observed that most of the Bulgarian sequences were interspersed with those introduced from various countries (Figure 3 and Figure 4). This pattern indicated the co-circulation of multiple SARS-CoV-2 lineages over time, likely linked to multiple importations followed by high local transmission, coinciding with a high number of infections (Figure 2).

To infer the number of viral imports and exports between Bulgaria and the rest of the world, we employed an ancestral location state reconstruction on the dated phylogeny (Figure 3 and Figure 4). To minimize sampling biases arising from the discrepancy in the number of available strains generated from different countries worldwide, we decided to aggregate the genomic data at the continent level, rather than at the country/regional level (Figure 3 and Figure 4). This approach allowed us to gain insights into the intricate interplay between imported cases and local transmission dynamics in Bulgaria. By tracking the flow of viral lineages across different geographic regions, we unraveled the patterns of viral dissemination and identified the key VOCs contributing to the pandemic’s progression within the country.

In the context of the Alpha and Delta variants, it was observed that the majority of imported introductions originated from Europe (76.8%) (Figure 3b,e). These introductions began in November 2020, before the implementation of restriction measures, at a time when the pandemic was rapidly progressing (Figure 3b,e). Up to December 2020, there were 21 estimated independent events related to the Alpha variant and 7 estimated independent events related to the Delta variant. Subsequently, a total of 114 introduction events took place during the enforcement of preventive measures up to April 2022 (Figure 3b,e), which was around the time when these measures began to be eased for the Alpha variant. For the Delta variant, most of the introduction occurred by the end of 2022, when the Omicron displacement happened, accounting for 411 introduction events estimated.

However, it is important to note that Bulgaria also played a notable role as a major exporter, especially to European countries and those in the Americas, emphasizing how the country’s connections and human mobility had differential impacts on the pandemic’s progression at both local and large scales. Specifically, our findings revealed that the total number of exportation events to Europe was 65.9% and 83.8% of the total (Alpha and Delta, respectively), while for the Americas, it was 15.9% and 6% (Alpha and Delta, respectively), and for Asia, it was 13.6% and 9.2% (Alpha and Delta, respectively), suggesting a dynamic pattern of transmission and dissemination in both directions (Figure 3c,f).

In our further investigation, we shifted our focus to the Omicron variants (BA.1, BA.2, and BA.5), which played a pivotal role in driving the subsequent epidemic peaks in the country (Figure 4). Our analysis revealed a significant number of imported introductions of these variants, with an estimated 352 (BA.1), 258 (BA.2), and 410 (BA.5) independent events. These introductions originated from both Europe (50.9%, 52.3%, and 54.4%, respectively) and the Americas region (42.3%, 43.4%, and 33.4%, respectively) (Figure 4b,e,h). Specifically, the European countries which were most involved were France for BA.1 and BA.2 and Germany for BA.2 and BA.5. Furthermore, we observed that for BA.1 and BA.2, no significant introductions were identified from Asia. However, for BA.5, approximately 12.2% of the introductions were traced back to Asian countries (Figure 4b,e,h).

Furthermore, we noticed that the total number of exportation events from Bulgaria to other continents was predominantly linked to European, Asian, and American countries (Figure 4c,f,i). For the BA.1 lineage, European countries (mostly France, Germany and the UK) received the largest share, accounting for 70.2% of the total events (Figure 4c). Similarly, for the BA.2 lineage, 62.9% of the exports were associated with European countries (mostly France, Germany and the UK), with an additional 23.3% towards Asian countries (mostly Republic of Korea) (Figure 4f). For the BA5 lineage, the exportation events were distributed across European countries (mostly France and Germany) (65.0%) and American (17.9%) countries (mostly USA), with a smaller proportion reaching Asian countries (mostly Japan) (16.3%) (Figure 4i).

These findings underscore the dynamic nature of SARS-CoV-2 lineages, revealing their remarkable ability to disseminate across diverse international destinations. The interconnectedness of global travel acts as a catalyst for the emergence and proliferation of SARS-CoV-2 variants, accentuating the imperative need for sustained global surveillance and collaborative efforts. Notably, European countries assumed a central role in propagating these variants into Bulgaria, while Asian and American nations equally experienced significant dispersion.

The fluidity of cross-border movement amplifies the risk of localized outbreaks and the establishment of novel transmission chains. Tracing the migration of viral lineages from Bulgaria to various continents yields invaluable insights that can inform targeted interventions. Genomic monitoring, data analysis, and timely public health actions are pivotal in mitigating the pandemic’s impact and implementing effective control strategies worldwide.

## 4. Discussion

The COVID-19 pandemic caused by SARS-CoV-2 had a profound impact on global health, social systems and the economy. Understanding the molecular epidemiology and lineage dynamics of this virus is crucial for devising effective public health strategies and mitigating the COVID-19 pandemic’s consequences. In this study, we conducted a comprehensive analysis of SARS-CoV-2 in Bulgaria over a three-year period, aiming to unravel the intricate interplay between viral evolution, transmission dynamics, and the country’s pandemic trajectory.

Our findings revealed a rich landscape of SARS-CoV-2 lineages circulating in Bulgaria, highlighting the importance of genomic surveillance in monitoring viral diversity and the emergence of VOCs. Through extensive genomic sequencing of SARS-CoV-2 in Bulgaria and data analysis, we were able to track the temporal and spatial distribution of viral lineages, providing insights into the patterns of viral transmission and possible sources of introduction into the country. This information is essential for understanding the factors influencing the dynamics of the pandemic as well as tailoring containment measures to specific regions and timeframes. One of the key observations from our study is the dynamic nature of SARS-CoV-2 lineages in Bulgaria. We identified multiple waves of viral transmission, each characterized by the dominance of distinct SARS-CoV-2 lineages and VOCs. The rise of VOCs, such as the Alpha, Delta and Omicron variants, coincided with significant increases in COVID-19 morbidity and mortality, underscoring the importance of monitoring and promptly responding to the emergence of potentially more transmissible or virulent variants. The Alpha wave was first displaced by the Delta variant, and similarly, the Omicron BA.1 variant initially displaced Delta after its global onset [22,23]. Moreover, BA.1 was the predominant lineage for a short period, while BA.2 prevailed for a longer duration. After several months of global dissemination, BA.5 became the most prevalent variant in mid-2022 [24]. The impact of vaccination efforts on the pandemic trajectory was evident in our analysis. While Bulgaria initially implemented control policies at the onset of the pandemic, the subsequent waves of infection posed significant challenges, leading to one of the world’s highest COVID-19 mortality rates. The low vaccination coverage in the country played a critical role in this trend, with immunization gaps allowing the virus to spread more effectively within susceptible populations [25]. The spread of recombinant variants in 2023 is an interesting phenomenon related to the evolution of SARS-CoV-2, which should be monitored with caution as the pandemic progresses [26].

Our viral exchange analysis allowed us to understand the dynamics behind SARS-CoV-2’s dissemination to and from the country. The interplay between international travel and viral transmission can lead to the emergence and dissemination of different variants, underscoring the importance of continuous monitoring and coordinated global efforts in combating the spread of infectious diseases. The observed proportions of exportation to various continents shed light on the potential pathways through which these lineages have spread globally. European countries received significant proportions of all lineages, suggesting a central role in the dissemination of these variants around the world. Countries in Asia and the Americas also had remarkable proportions of these viruses, indicating their widespread distribution in different parts of the world. It is important to note that the international transfer of SARS-CoV-2 variants can significantly impact the global epidemiological landscape. As variants cross borders, they can contribute to localized outbreaks or even lead to the establishment of new transmission chains in different regions. Therefore, collaboration and data sharing between countries is crucial to better understand and respond effectively to the evolving dynamics of the pandemic. The identification and tracking of the viral lineages originating from Bulgaria and spreading to different continents provide critical insights into the potential routes of cross-border transmission. By understanding these patterns, public health authorities can devise targeted interventions and containment measures to limit the further spread of SARS-CoV-2 viruses. Continuous vigilance, data analysis, and timely public health actions are key to mitigating the impact of the pandemic and protecting local populations as well as global health. As the virus continues to evolve, it is vital to stay proactive in monitoring and responding to new variants to ensure effective control and prevention strategies worldwide.

In conclusion, this study offers critical insights into the evolution of SARS-CoV-2 and its impact on the trajectory of the pandemic in the country. The results underscore the significance of genomic surveillance in guiding evidence-based public health interventions, such as vaccination strategies, containment measures, and outbreak response. The comprehensive understanding of viral diversity and transmission dynamics obtained from this analysis can serve as a blueprint for similar studies in other regions, contributing to the global effort to combat the challenges of the COVID-19 pandemic. To achieve the effective control and mitigation of the pandemic, sustained genomic surveillance and collaboration between public health agencies at the national and international levels are essential.

## Data Availability

All genome sequences from Bulgaria and the other countries used in this study are freely available in the GISAID databases. In addition, the dataset used in this study is available upon request from the corresponding author.
